# Journals that accept case reports 2025

**DOI:** 10.5195/jmla.2026.2436

**Published:** 2026-10-01

**Authors:** Terri Gotschall, Elizabeth Irish, Emily Hannum, Kryslynn Collazo, Alyssa Snyder, Jacquelyn Simington

**Affiliations:** 1 tgotschall@rollins.edu, AI Librarian, Rollins College, Winter Park, FL; 2 irishe@amc.edu, Professor of Medical Education, Schaffer Library of Health Sciences, Albany Medical College, Albany, NY; 3 emily.hannum@ucf.edu, Research Services Librarian, University of Central Florida College of Medicine, Orlando, FL; 4 kcollazo1@usf.edu, Library Services Supervisor, Valencia College, Orlando, FL; 5 snydera6@amc.edu, Fourth Year Medical Student, Albany Medical College, Albany, NY; 6 simingj@amc.edu, Fourth Year Medical Student, Albany Medical College, Albany, NY

**Keywords:** Publishing, case reports, scholarly communication

## Abstract

**Background:**

For medical students and residents writing and publishing a case report is a common entry point into scholarly publishing. However, finding a journal that accepts case reports can be challenging. This project updates the 2023 list of journals that accept case reports. Shared on the Open Science Framework (OSF), the original list is a highly used, openly available list of English-language, MEDLINE-indexed journals that publish case reports, providing librarians and authors with a vetted, current open resource for discovering journals.

**Case Presentation:**

A list of 2,199 journals was extracted from PubMed using the same method from 2023. The methodology was updated to exclude journals that rarely publish case reports (n=721) resulting in 1,478 journals to review for inclusion on the updated list. The team excluded 290 titles due to ceased publication (n=1), submission restrictions (n=1), non-English publication (n=13), lack of MEDLINE indexing (n=4), and not publishing case reports (n=271). The final list includes 1,188 journals. This is an additional 160 journals over the 2023 list. Nearly half of eligible journals (49%) offered free-to-author publishing options, either through traditional publishing models, open access that did not charge article processing fees, or through hybrid publishing models.

**Conclusion:**

The Journals that Accept Case Reports list is an open resource available on the Open Science Framework and serves as a tool to support librarians and authors in identifying appropriate journals for publishing a case report.

## BACKGROUND

Research and publishing are actively promoted as essential for undergraduate medical education [1]. The transition of the United States Medical Licensing Examination (USMLE) Step 1 to pass/fail scoring from numerical scoring in 2022 shifted the residency application landscape, increasing the importance of research and scholarship participation for medical students and residency program directors. This is particularly true for medical students looking to match with more competitive specialties and higher-tiered programs [2]. As a result, accessible forms of academic productivity are crucial early in training, and case reports are often the introduction to publishing for medical students, residents, and junior faculty [3].

Despite their lower position in the evidence hierarchy, case reports continue to have a role in the advancement of medical knowledge [4]. Unique cases can highlight successful diagnosis and care of not only novel diseases and newly discovered variants, but also new approaches to standard of care. Their findings can be the basis for future research studies, thus promoting higher levels of research and evidence synthesis [5].

The medical education value of case reports goes beyond their contribution to medical knowledge. Case reports have been identified as one of the first opportunities for medical scholars to actively engage in researching, constructing, editing, and submitting a manuscript. As part of the publication process, students and residents practice critical thinking, team work, and medical writing skills within a short time period [6,7]. Additionally, unlike other article types, case reports usually are institutional review board (IRB) exempt. They typically do not require statisticians, long-term follow up, or exhaustive research while juggling long hours in a clerkship or residency program.

The first list of journals that accept case reports was published in 2016 and included 160 journals [8]. In 2023, a second list of journals that accept case reports was published, and included 1,028 journals [9]. In 2024, Gaggero published a list of 39 open-access, no fee journals discoverable in PubMed and Scopus that publish image reports [10]. These lists demonstrate a need to support authors in finding journals that publish a specific article type.

The 2023 Journals that Accept Case Reports (JACR) list has been a valuable resource for the promotion of undergraduate medical research and a highly visited resource [11]. Since the list was posted, it has been viewed over 8,000 times and the authors are aware of the list being included on 11 library websites. With the shift toward open access publishing, maintaining an updated list is critical for recognizing journals whose APC (article processing charge) could be prohibitive for students lacking access to dedicated research funding [12]. One of the limitations of the project is the changing publication landscape, making the list less accurate over time. Therefore, this project is being updated to make sure the list remains useful and up to date.

This project updates the 2023 list of journals with the objective to create an extensive, but not an exhaustive, list of journals that are indexed in MEDLINE, are published in English, and actively publish case reports. This vetted list is being updated to help medical students or resident authors, and the librarians who support them, find journals that accept case reports. The list is freely disseminated on the Open Science Framework.

## CASE PRESENTATION

### Journal Search

A list of journals was extracted from PubMed by conducting an all field search for (“case report*”). The title/abstract field was not used, as it excluded journals that published case reports without abstracts or “case report” in the title. The limiters: article type case reports, English, and MEDLINE were applied. The same rationale from the previous study was used for selecting the limiter of MEDLINE. This includes the indexing requirements of journals to be included in MEDLINE and the ability to search MEDLINE from multiple databases and search engines [5]. To identify journals that were currently publishing case reports, date limiters were used to pull records from July 1, 2024 to June 30, 2025. Because PubMed’s export limit is 10,000 records, records were pulled in six batches July 1, 2024 to August 31, 2024 (n=9,459), September 1, 2024 to October 31, 2024 (n=8,542), November 1, 2024 to December 31, 2024 (n=6,608), January 1, 2025 to February 28, 2025 (n=6,976), March 1, 2025 to April 30, 2025 (n=5,819), May 1, 2025 to June 30, 2025 (n=4,549). This search resulted in 41,953 results. The results were exported as csv files and compiled in Microsoft Excel.

### Journal Selection

Because journal title abbreviations are a controlled vocabulary in PubMed, a pivot table was created in Excel to remove duplication and to determine the number of case reports published in each journal. As a result, 2,199 unique journal titles were identified. This is an increase in the number of journal titles to vet from the 2023 list (n=1,874). One of the issues with the 2023 list was the inclusion of journals that rarely publish case reports. The 2023 final list included 202 journals that stated on their website that they accepted case reports but only under certain circumstances or rarely accepted case reports. Since the 2025 list is for authors who are searching for a journal that is actively seeking to publish a case report, journals that rarely publish were excluded. Therefore, journals were included if they had published three or more case reports between July 1, 2024 and June 30, 2025. Journal titles that had only published one or two case reports were excluded (n=721). Resulting in 1,478 unique journal titles.

The 2025 list of journals to vet was compared to the 2023 list of journals to vet. The 2025 list of journals to vet included 1,224 titles that were included on the 2023 list, and an additional 254 new journal titles that were not vetted in 2023. There were 650 journals on the 2023 list to vet that were not included on the 2025 list to vet. These were compared to the final results of the 2023 list, 446 of these titles either did not accept case reports or only accepted case reports under certain circumstances. The remaining 204 journals were compared to the 2025 list that were excluded for only publishing one or two articles, 108 of the 2023 journal titles had only published one or two case reports during the 2024–2025 time period. Further supporting the decision to up-to-date the list, ninety-six journals who had published a case report in 2023, had not published a case report during the 2024–2025 time period.

### Data Collection

The following data points were collected using a Microsoft Form: Journal title abbreviation, full journal title, MEDLINE inclusion status, Broad Subject Term (specialty), does the journal accept case reports, and publishing model.

The National Library of Medicine (NLM) assigns broad subject terms to journals that are indexed in MEDLINE providing a controlled vocabulary of 130 specialty areas [13]. Journals may be assigned multiple broad subject terms and all terms for a journal were recorded.

The PubMed records for journal titles only include the abbreviated title. The team used the NLM catalog to search for the journal title abbreviation. From the journal record the full journal title, and the broad subject terms were obtained. In addition, the inclusion in MEDLINE was also confirmed, and the electronic links were used to navigate to the journal websites. If a link was broken, the team searched the internet for the journal title. From the journal’s website, team members navigated to the author’s guideline to confirm the journal accepted case reports, to collect the link to the author guidelines, and to collect the publishing model information (subscription with no author fees, subscription with author fees, open access with no fees, open access with fees, or hybrid).

## RESULTS

Of the 1,478 journal titles reviewed, 1,188 journals were found to accept case reports, covering 111 subject areas, 290 titles were excluded: one journal that had ceased publication, one journal that did not accept submissions, 13 journals whose websites were not available in English (these website did not have an option to translate into English and an attempt to use Google’s translate did not result in a usable translation), four journals that were not indexed in MEDLINE, and 271 did not accept case reports, or only accept case reports under certain circumstances, for example the website used phrases such as “case reports are strongly discouraged,” “case reports are unlikely to be accepted” or “case reports are of low priority”. These journals still published three case reports, so they were on the list of journals to vet, however, it is unclear as the circumstances in which they accept and publish case reports.

During the JACR 2023 review process, 803 journals reviewed did not accept case reports. In comparison, in 2025 only 271 journals reviewed did not accept case reports. The new method of eliminating journals that rarely publish case reports, may have improved the accuracy of discovering journals that are actively publishing case reports. Sixty-six journals that were included on the 2023 list that were reviewed for 2025 no longer accept case reports, discourages case report submissions, or suggested sending case reports to their “partner journal”.

### Publishing Models

Nearly half of journals, 49% (n=549) offer a free for authors publishing model (Table 1).

**Table 1:** Publishing models.

Publishing Model	Total	Percentage
Open Access (charges author fees)	262	22%
Open Access (no author fees)	89	7%
Optional Open Access (author chooses subscription may be charged fees or is charged fees for open access)	349	29%
Optional Open Access (Author chooses subscription, no fees, or is charged fees for open access	414	35%
Subscription (charges author fees)	28	2%
Subscription (charges no author fees)	46	4%
Total	1,188	

The publishing models remained relatively consistent between 2023 and 2025.

**Table 2:** Comparison of publishing models from 2023 to 2025.

Publishing Models	2023 Total	2023%	2025 Total	2025%
Subscription	144	14%	74	6%
Hybrid	581	57%	763	64%
Open Access	303	29%	351	30%

## DISCUSSION

Case reports continue to be an introductory publication avenue for entry level healthcare providers. Their educational value has been identified as providing students and residents with the opportunity to practice critical thinking, teamwork, and medical writing skills [6,7,14]. Jha et al’s single-institution study on the value of case reports found fourth year medical students perceived case reports as improving their scientific writing, presentation and critical thinking skills [15]. Packer et al. studied case reports published by medical students in the Veterans Administration hospital system between 2008 and 2015. Based on the team’s data analysis and student feedback, they propose five educational benefits: “(1) observation and pattern recognition skills; (2) hypothesis-generating skills; (3) understanding of patient-centered care; (4) rhetorical versatility; and (5) use of the case report as a rapidly publishable ‘mini-thesis,’” [6]. To build on Packer’s benefits, case reports can serve as proof of concept. Albrecht et al examined 103 case reports and case series published in the Lancet between 1996–1997. Of the 103 studied, 96 were cited an average of 17 times and 22% led to further clinical research [16]. The educational value of case reports rests in their being viewed as the first stage of research, not the last.

As research productivity becomes increasingly prominent in residency selection, identifying accessible and feasible avenues for scholarly output among medical students becomes essential. Being able to identify journals that accept case reports facilitates the scholarly process for students who are building their scholarship for their residency applications. Websites catering to medical students eager to make their ERAS application stand-out to residency program directors, promote case reports as one of the first steps towards building a publication portfolio [17]. One or two case reports published in a quality journal can demonstrate to program directors that a student can identify a unique case, work as part of team, and see a project through from the beginning to the end [18]. In late 2025, the Association of American Medical Colleges (AAMC) announced changes to the Electronic Residency Application Service (ERAS), which puts more focus on peer-reviewed scholarly work [19]. As MEDLINE journals, the titles included on the list are peer-reviewed.

Distinctions between open access and subscription-based journals have important implications for students, residents, and fellows as the rising costs of APCs are a barrier to publication [20]. Booth et al quantified the staggering cost associated with APCs, including monetary implications such as shifts to non-peered reviewed preprints as authors attempt to avoid rising costs of publishing [21]. Librarians, who advise authors, can utilize the publishing model aspect of the list when helping authors to find journals that do not charge authors for publication.

Despite fewer journals explicitly refusing case reports in 2025 compared to 2023, this does not equate to increased accessibility. Some journals that were vetted did not accept case reports, instead offering to transfer authors' case report submissions to “partner journals” which were open access journals that charged article processing fees, not indexed in MEDLINE, and cited less often [20,22]. As case reports are non-funded publications this tactic results in financial barriers that disproportionately affect students, residents, and early career clinicians who rely on case reports as an entry point into scholarly publishing [21].

In the current publishing landscape, predatory journals continue to be a challenge. Their schemes are so pervasive, that in 2025 an editorial written by editors and members of the International Committee of Medical Editors (ICJME) was published simultaneously in 14 journals [23]. The editorial discussed how predatory journals engage in aggressive solicitation, false promises related to publication timeline, and lack transparency about cost and fees. Early career scholars may not yet have the experience and knowledge needed to discern the credibility of a journal. Due to concerns of predatory journals, and the goal of this list to support early career scholars, the JACR list limits journals to those indexed in MEDLINE.

To put the MEDLINE subset selection process into perspective, only 8% (n=11/145) of the journals evaluated for MEDLINE inclusion in 2025 met the scientific review standards [24]. Providing the authors with confidence, but not a guarantee, that the JACR list promotes legitimate journals. MEDLINE journals are considered the gold standard of vetted publications with measurable evaluation criteria, including the use of CARE for case reports [25]. The CARE Guidelines was adopted at the 2013 International Congress on Peer Review and Biomedical Publication to strengthen the completeness and transparency of case reports [26]. In part due to the growth of case report journals, there have been calls to update CARE guidelines to improve the quality of submissions [27].

The JACR list is freely available on the Open Science Framework under a creative commons license. This tool can help librarians and authors identify appropriate journals for publishing case reports in an increasingly complex and cost-driven publication landscape.

## LIMITATIONS

Using the MEDLINE filter may have excluded some journals that are actually indexed in MEDLINE, just as the list to vet included some journals that were not currently indexed in MEDLINE. As with the 2023 list, this list will also become outdated. Therefore, this list should be used as a starting point for discovering journals that accept case reports, and not as a definitive list.

## Data Availability

Journals that Accept Case Reports 2025 is available on Open Science Framework (OSF), https://osf.io/fx28d under the CC-BY Attribution 4.0 International Creative Commons license.
